# Multi-criteria decision analysis: of politics, policy entrepreneurs, and processes

**DOI:** 10.1186/s12962-018-0131-x

**Published:** 2018-11-09

**Authors:** Victoria Y. Fan

**Affiliations:** 1 Office of Public Health Studies, Myron B. Thompson School of Social Work, University of Hawai‘i at Mānoa, 1960 East-West Road, Biomed D204, Honolulu, HI USA; 2 François-Xavier Bagnoud Center for Health and Human Rights and the Department of Global Health & Population, Harvard T.H. Chan School of Public Health, 651 Huntington Ave, Boston, MA USA

**Keywords:** Health technology setting, Priority setting, Political economy, Transparency, Accountability, Planning and policy

## Abstract

Multi-criteria decision analysis (MCDA) has the potential to increase the consistency, transparency and rigour with which these criteria inform decisions. Political context is relevant not only as a motivation for turning to MCDA but also the context in which MCDA can be successfully implemented. A policy entrepreneur can spearhead the creation of a process to carry out MCDA and can help to create and build the capacity of a public institution that observes and convenes this process, an institution that has legal authority to carry out such a function.

Multi-criteria decision analysis (MCDA) is proposed as part of health technology assessment (HTA) because it offers a means to formally consider a more comprehensive set of benefits than conventional HTA methods, as indicated by several papers in this special issue. While MCDA has the potential to make a contribution to HTA, creating the process to carry out and use MCDA will perhaps need a policy entrepreneur and appropriate institution and governing process for setting priorities.

HTA is multi-criteria in its nature [1]. Where a more narrow perspective has been proposed, it has been met with resistance. One notable example of such single-factor decision-making was the Oregon Medicaid Priority-Setting Project in 1991 [2], which attempted to use only cost-effectiveness to determine a benefit package, to the outcry of many observers and led to pushback against such narrow use.

In other instances, excessive focus on a single criterion has been used to obscure the real reason for decisions. For example, in the early stages of the HIV epidemic, there was considerable policy resistance against providing anti-retroviral therapy for people in developing countries. One of the most commonly cited reasons included the high cost and the lower cost-effectiveness compared to other interventions. Fortunately, other reasons ultimately trumped those arguments, as advocates garnered greater political commitment, overcame stigma, racism, and discrimination, and advocated for a concern for health equity [3, 4]. Later on, treatment became very cost-effective as prices of drugs went down and the cost-effectiveness argument became irrelevant, as antiretroviral therapy became as cost-effective as oral rehydration therapy [5]. Clearly, in this case, cost and cost-effectiveness were not the only reasons behind the resistance against expanding treatment.

Thus, the motivation for adopting MCDA is less about broadening the criteria being considered in HTA, as these criteria are probably already being considered. Rather MCDA can increase the consistency, transparency and rigour with which these criteria inform decisions. This is a critique of decisions made at the discretion of political powerful individuals and decision-makers. In my own past work on assessing the allocation of HIV funding across countries, explaining why HIV funds have been distributed across countries the way they are is not easy, suggesting leeway for decision-makers [6]. Decision “analysis” is a technocrat’s hope, but decisions are made by those with power. The role of political discretion in deciding which interventions to include in a benefit package is perhaps not well understood, especially in the United States. How does an insurance company decide how to design their benefit package? How does a government body decide which set of health care benefits ought to be deemed essential or mandated by all health insurance plans? In this context, arguments for MCDA often evoke the concept of accountability for reasonableness, which identifies the features of an accountable and transparent process for public deliberation [7].

However, the political context is not just relevant as the motivation for turning to MCDA. It also represents the context in which MCDA needs to be implemented. Understanding the political economy of health care—health care decisions, health care reform—is essential. There are a variety of principles, methods or approaches that can help make decisions more predictable, more systematic, and more—(choose your favorite principle, e.g. equitable or efficient or whatever). But as important as the process is a cognizance of, if not a familiarity with, the politics of creating such a process for MCDA. How can such a process be created? How can the decisions of such an accountable process in fact have “teeth” and be adopted?

The literature on political economy and agenda setting points to the importance of so-called policy entrepreneurs in bringing about such changes. They take advantage of timing, budget cycles, knowledge of rules, regulations, policies and procedures, as well as a firm commitment to a common goal, in this case, creating a process for producing fairer decisions for priority setting [8]. Policy novices could use political analysis to help to decide whether a particular policy can and may be adopted and implemented, or even whether such an accountable and fair process can be created [9]. The policy entrepreneur would be needed to spearhead the creation of the process to carry out MCDA. That individual would also need to help establish the rules for creating the process for MCDA. These rules include who can participate, whether those persons have declared conflicts of interest, when, where, and how decisions will be made, and so on. In other words, the policy entrepreneur needs to navigate the political process to create the MCDA process. As MCDA becomes more widely used, more research and cases are needed that document and understand the political challenges and how these have been overcome to implement MCDA.

Finally, creating a process to carry out MCDA may be insufficient. There may be a need for a public institution that observes and convenes this process, an institution that has legal authority to carry out such a function. Countries around the world are creating such authoritative priority-setting institutions, such as the UK National Institute for Clinical Excellence (NICE), the Thai Health Intervention and Technology Assessment Program (HITAP), and others [10]. Capacity of such institutions to carry out such methods is growing and with greater international support, such as through the International Decision Support Initiative (IDSI) and others. More work is needed to understand the extent and success to which such institutions integrate and use a variety of health technology assessment methods and approaches, including MCDA, and specifically the challenges of building institutions and developing human resources and build capacities.

In short, creating the process for MCDA needs political savvy, perhaps spearheaded by a policy entrepreneur. But what may well be needed is a separate institution to govern this process for MCDA or other approach using multiple criteria for setting priorities.
